# The impact of sarcopenia on the efficacy of PD-1 inhibitors in non-small cell lung cancer and potential strategies to overcome resistance

**DOI:** 10.3389/fphar.2024.1377666

**Published:** 2024-07-19

**Authors:** Zhenchao Liu, Tianxiang Lei, Yunliang Guo, Chongwen Zheng

**Affiliations:** ^1^ School of Pharmacy, Qingdao University, Qingdao, Shandong, China; ^2^ Institute of Integrative Medicine, Qingdao University, Qingdao, Shandong, China; ^3^ Department of Neurology, The 2nd Affiliated Hospital of Fujian University of Traditional Chinese Medicine, Fuzhou, China

**Keywords:** sarcopenia, non-small cell lung cancer, programmed cell death protein-1, immune checkpoint (ICIs), muscle

## Abstract

Recent studies have revealed that sarcopenia can adversely affect the efficacy of PD-1 inhibitors in the treatment of non-small cell lung cancer (NSCLC). PD-1 inhibitors are immune checkpoint inhibitors widely used in the treatment of various cancers. However, NSCLC patients may have poorer outcomes when receiving PD-1 inhibitor treatment, and sarcopenia may affect the efficacy of PD-1 inhibitors through immune and metabolic mechanisms. In this article, we summarize the reported negative impact of sarcopenia on the effectiveness of PD-1 inhibitors in the treatment of NSCLC in recent years. Based on existing research results, we analyze the possible mechanisms by which sarcopenia affects the efficacy of PD-1 inhibitors and discuss possible strategies to address this issue. This could help to understand the impact of sarcopenia on the treatment of PD-1 inhibitors and provide more accurate expectations of treatment outcomes for clinicians and patients. Additionally, we present tailored intervention strategies for sarcopenic patients undergoing PD-1 inhibitor therapy, aiming to optimize treatment efficacy and enhance patient quality of life. Nevertheless, further research is warranted to elucidate the mechanisms through which sarcopenia impacts PD-1 inhibitors and to identify more efficacious intervention approaches for improving the effectiveness of PD-1 inhibitor treatment in sarcopenic patients.

## 1 Introduction

Non-small cell lung cancer (NSCLC) is the most prevalent form of lung cancer, accounting for approximately 80%–85% of all cases (Yang et al., 2020). The development of NSCLC is driven by genetic mutations and alterations in the lining of lung cells. These mutations can lead to uncontrolled growth and division of lung cells, forming tumors. Common gene mutations associated with NSCLC include In review epidermal growth factor receptor (EGFR) and anaplastic lymphoma kinase (ALK) gene mutations (Forsythe et al., 2020). Treatment options for NSCLC currently include surgery, radiation therapy, chemotherapy, targeted therapy, and immunotherapy. Among these, immune checkpoint inhibitors (ICIs), specifically programmed death-1 (PD-1) inhibitors, have demonstrated significant success in treating advanced NSCLC (Yang et al., 2020; Flores et al., 2021). However, emerging evidence suggests that the presence of sarcopenia, a condition characterized by muscle loss, may impact the effectiveness of PD-1 inhibitors in NSCLC patients.

Sarcopenia is a disease characterized by a decrease in skeletal muscle mass, strength, and physical performance. In 2010, the European Working Group on Sarcopenia in Older People (EWGSOP) officially defined sarcopenia as “a syndrome characterized by progressive and generalized loss of skeletal muscle mass and strength” (Cruz-Jentoft et al., 2010a). The decline in muscle strength and physical performance reduces the functional activity capacity of patients. Additionally, the loss of skeletal muscle deprives patients of protection and dynamic support, increasing their risk of falls, fractures, and disability compared to the general population (Petermann-Rocha et al., 2022). The concept of sarcopenia was first proposed by Rosenberg in 1989 (Rosenberg, 1989). In 1998, Baumgartner et al. (Janssen et al., 2004) defined sarcopenia and used dual-energy X-ray absorptiometry (DXA) for its diagnosis. They introduced the skeletal muscle mass index (SMI) as a reference index for sarcopenia, SMI = the muscle mass of the limbs measured by DXA/the square of height. Sarcopenia is defined as SMI below two standard deviations of healthy adults of the same gender aged 18–40 years, or SMI below 7.26 kg/m^2^ in males and 5.45 kg/m^2^ in females. Subsequently, the European Working Group on Sarcopenia in Older People (EWGSOP) (Cruz-Jentoft et al., 2010b; Cruz-Jentoft et al., 2019), the International Working Group on Sarcopenia (IWGOS) (Fielding et al., 2011), and the Asian Working Group for Sarcopenia (AWGS) (Chen et al., 2014; Chen et al., 2020) proposed corresponding diagnostic criteria for sarcopenia, further standardizing its definition and diagnosis (See Table 1). The assessment of sarcopenia usually involves the comprehensive evaluation of both skeletal muscle mass and physical function (grip strength and walking speed). Various methods are commonly used to assess skeletal muscle mass, including DXA, bioelectrical impedance analysis (BIA), computed tomography (CT), and magnetic resonance imaging (MRI). CT and MRI are considered the gold standards for assessing skeletal muscle mass, often using the L3 vertebra as a landmark for measurement. CT images evaluate muscle mass by measuring tissue attenuation, while MRI provides more accurate measurements of muscle tissue in specific regions. However, due to their high cost and limitations, DXA and BIA are more commonly used methods. DXA uses dual-energy X-rays to measure whole-body and regional body composition, offering the advantages of simplicity, minimal radiation exposure, and convenience. BIA estimates muscle mass by measuring electrical impedance and is characterized by its simplicity, non-invasiveness, and affordability.

**TABLE 1:** Diagnostic criteria and key thresholds for sarcopenia.

Diagnostic criteria	Skeletal muscle Mass index	Grip strength (kg)	Gait speed (m/s)
EWGSOP 2010 (Cruz-Jentoft et al., 2010b)	SMI (kg/m^2^) DXA male<7.0, female<6.0	male<27, female<16	≤0.8 (4 m)
EWGSOP 2018 (Cruz-Jentoft et al., 2019)	SMI (kg/m^2^) DXA male<7.0, female<6.0	male<27, female<16	≤0.8 (4 m)
IWGS (Fielding et al., 2011)	SMI (kg/m^2^) DXA male<7.23, famle<5.67	-	<1.0
AWGS 2014 (Chen et al., 2014)	SMI (kg/m^2^) DXA male<7.0, female<5.4 BIA male<7.0, female<5.7	male<20, female<15	<1.0 (6 m)
AWGS 2019 (Chen et al., 2020)	SMI (kg/m^2^) DXA male<7.0, female<5.4 BIA male<7.0, female<5.7	male<20, female<15	<1.0 (6 m)

EWGSOP, european working group on sarcopenia in older people; IWGS, international working group on sarcopenia; AWGS, asian working group for sarcopenia; FNIH, foundation for the national institutes of health; SSCWD, society on sarcopenia, Cachexia and Wasting Disorders; SMI, skeletal muscle mass index; BMI, body mass index; DXA: Dual Energy X.

Sarcopenia is highly prevalent in cancer patients and is associated with increased mortality. Recent studies have shown that sarcopenic patients may experience poorer overall survival (OS) and progression-free survival (PFS) compared to non-sarcopenic patients when treated with PD-1 inhibitors. Comprehending the influence of sarcopenia on the efficacy of PD-1 inhibitors is essential for identifying strategies to mitigate this impact and enhance treatment outcomes in patients with NSCLC.

In this review, we aim to comprehensively explore the influence of sarcopenia on the efficacy of PD-1 inhibitors in NSCLC and discuss potential strategies to mitigate its deleterious effects. Through the clarification of underlying mechanisms and the proposal of effective interventions, this review strives to contribute to the advancement of personalized strategies that optimize the therapeutic benefits of PD-1 inhibitors in NSCLC patients, irrespective of their sarcopenia status. Ultimately, these endeavors may result in enhanced clinical outcomes and improved quality of life for NSCLC patients undergoing PD-1 inhibitor therapy.

## 2 The relationship between sarcopenia and NSCLC

Sarcopenia was considered a muscle aging syndrome primarily affecting the elderly, especially those over 65 years old. Its prevalence increases with age and is associated with age-related physiological decline in skeletal muscle mass and function (Punga and Ruegg, 2012). Nevertheless, subsequent research has unveiled that sarcopenia is not exclusive to the elderly and can manifest in younger populations as well. As a result, the 2018 consensus on sarcopenia by EWGSOP recognizes that while sarcopenia is prevalent among the elderly, it can also occur in younger individuals (Cruz-Jentoft et al., 2019). Additionally, several chronic diseases, including malignant tumors, type 2 diabetes mellitus, liver cirrhosis, rheumatoid autoimmune diseases, and cardiovascular diseases, can contribute to the development of secondary sarcopenia. The prevalence of secondary sarcopenia surpasses that of primary sarcopenia and significantly impacts patients’ prognosis and rehabilitation outcomes (Liao et al., 2021; Supriya et al., 2021).

Sarcopenia is a multifactorial condition characterized by the impairment of muscle mass and function, influenced by various factors through distinct signaling pathways. Among these factors, hindered protein synthesis plays a significant role in the development of sarcopenia. Aging, malnutrition, and inadequate protein intake contribute to the reduced capacity for muscle protein synthesis. Key signaling pathways involved in muscle protein synthesis include the mechanistic target of rapamycin (mTOR) and protein kinase B (PKB or Akt). The mTOR pathway promotes muscle protein synthesis by activating mTOR complex (mTORC), while the Akt pathway activates mTOR and regulates other protein kinases to control protein synthesis and degradation (Stitt et al., 2004; Zanchi and Lancha, 2008; Rondanelli et al., 2016; Ji et al., 2020). Additionally, sarcopenic muscles exhibit elevated expression of forkhead box O3 (FOXO3). FOXO3 promotes protein degradation by activating genes such as Atrogin-1 and MuRF1. It can also activate apoptosis-related genes, including BNIP3, thereby promoting cell apoptosis. Conversely, reducing the expression of FOXO3 inhibits cell apoptosis, activates satellite cells, and promotes their differentiation into muscle cells (Oh et al., 2023). Furthermore, changes in hormone levels also contribute to sarcopenia. With advancing age, levels of growth hormone (GH) and testosterone decline. GH and testosterone promote muscle growth and maintenance by activating signaling pathways such as phosphoinositide 3-kinase/protein kinase B (PI3K/Akt) and mTOR. They inhibit the activity of FOXO transcription factors, reduce protein degradation and autophagy, thereby facilitating muscle synthesis and maintenance. In addition, chronic low-grade inflammation is another significant contributor to sarcopenia (Fuh and Bach, 1998; Kenny et al., 2003; Hosoi et al., 2023). As age increases, concentrations of inflammatory factors such as interleukin-6 (IL-6) and tumor necrosis factor-alpha (TNF-α) in the body rise. These inflammatory factors promote muscle protein degradation by influencing mTOR and FOXO signaling pathways, leading to sarcopenia (Wang et al., 2014; Hardee et al., 2018; Liu L. et al., 2021; Yang B. et al., 2022). Furthermore, oxidative stress is also a crucial mechanism underlying sarcopenia. As age increases, cells generate more reactive oxygen species (ROS) at rest, resulting in increased oxidative stress. Moderate levels of ROS can stimulate muscle development and adaptive responses, but excessive ROS levels inhibit protein synthesis and induce apoptosis and autophagy in muscle cells, ultimately leading to sarcopenia (Wang et al., 2019; Chi et al., 2022). In summary, sarcopenia is driven by various factors, including hindered protein synthesis, alterations in hormone levels, chronic low-grade inflammation, and oxidative stress. These factors influence muscle growth and degradation through the regulation of signaling pathways such as mTOR, Akt, and FOXO. A deep understanding of these mechanisms is crucial for the development of preventive and therapeutic strategies for sarcopenia.

Muscle-related changes are considered to be NSCLC-related factors, which may result from malnutrition, inflammatory responses, and tumor metabolism. These factors may contribute to reduced Akt and mTOR activity in skeletal muscle tissue of cancer cachexia patients, resulting in decreased muscle protein synthesis, impaired muscle growth and repair, and increased muscle protein degradation. Reduction in muscle mass and weakness not only have a negative impact on the physiological status of the patients but also affect the function of the immune system and anti-tumor response, which are associated with poorer survival rates and higher risk of complications (Khaddour et al., 2020; Takahashi Y. et al., 2021; Mangano et al., 2022; van de Worp et al., 2023).

Existing studies have shown that sarcopenia is an independent predictive factor for PFS and OS in NSCLC patients (Ding et al., 2023; Yuan et al., 2023). Sarcopenia not only affects the physical function and quality of life of patients but also has adverse effects on various quality outcomes for NSCLC patients (Karaman et al., 2023). Hasenauer et al. observed 401 early-stage NSCLC patients who underwent video-assisted thoracoscopic anatomical lung resection and found that 92 patients had sarcopenia (Hasenauer et al., 2023). The analysis results showed that these patients had a significantly higher postoperative complication rate and prolonged hospital stay. Studies by Nakamura et al. and Ushitani et al. both showed that sarcopenia is an independent factor influencing the prognosis of NSCLC (Icard et al., 2018; Ushitani et al., 2022). Watanabe et al. conducted a retrospective analysis of patients with stage II/III NSCLC who underwent neoadjuvant chemoradiotherapy and surgery, suggesting that muscle assessment before neoadjuvant chemoradiotherapy may help determine the optimal treatment strategy and appropriate nutritional and exercise interventions (Watanabe et al., 2023). The studies by Deng et al. and Kawaguchi et al. indicated that muscle damage and weakness caused by sarcopenia may also be predictive factors for recurrence after surgical resection in NSCLC patients (Deng et al., 2019; Kawaguchi et al., 2021). In a study by Kikuchi et al. analysis of 74 NSCLC patients with concomitant interstitial lung disease who received chemotherapy revealed that sarcopenic patients had significantly shorter OS (Kikuchi et al., 2022). In conclusion, sarcopenia has an adverse impact on postoperative complication rates, hospital stay, and prognosis in NSCLC patients, and muscle assessment can guide the selection of treatment strategies and interventions.

## 3 The mechanism of action of PD-1 inhibitors and their clinical applications in NSCLC

PD-1 and its ligand (PD-L1) inhibitors are a type of ICIs widely used in the treatment of various cancers, including melanoma, NSCLC, and renal cell carcinoma. They have demonstrated significant therapeutic effects and are considered a significant breakthrough in immunotherapy so much so that they became a hot topic in cancer immunotherapy research in recent years (Sasikumar et al., 2021).

PD-1, a 40 kDa transmembrane protein belonging to the B7 family, is predominantly expressed on activated immune cells like T cells, B cells, and Natural Killer (NK) cells. As an immune checkpoint molecule, PD-1 is part of a group of proteins responsible for regulating immune system activity. These proteins play a crucial role in modulating immune cell functions and immune responses (Kalim et al., 2020). PD-L1 and PD-L2 are the two primary ligands that bind to PD-1. PD-L1 is primarily expressed on tumor cells and various bone marrow cells, while PD-L2 is primarily found on activated dendritic cells and macrophages. Both ligands play important roles in suppressing anti-tumor immune responses. By interacting with PD-1, they inhibit T cell signaling pathways, proliferation, survival, and cytokine production, thereby preventing excessive activation and occurrence of autoimmune reactions. During the escape phase, cancer cells can evade the immune system attack by producing PD-L1 to bind with PD-1, thus, inhibiting T cell activity (Kalim et al., 2020; Liu J. et al., 2021). Therefore, PD-1 is regarded as an important immune regulatory molecule and has become a critical target in tumor immunotherapy (Yang et al., 2020).

**FIGURE 1:**
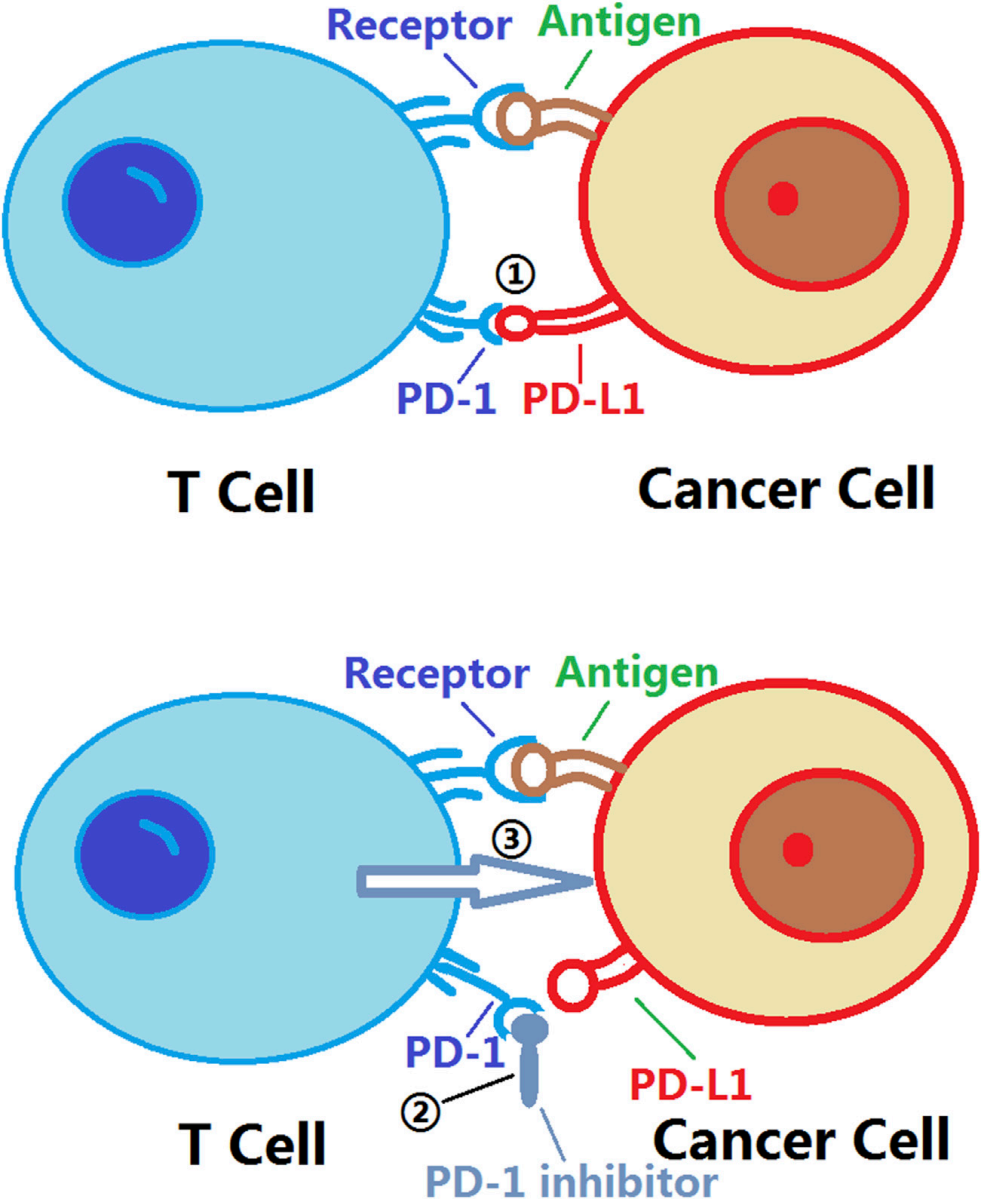
Mechanism of action of PD-1 inhibitors. PD-1: Programmed Cell Death Protein 1; PD-L1:Programmed Death-Ligand 1. ① The binding of PD-L1 to PD-1 triggers an inhibitory signal that suppresses the activity and function of T cells; ② The binding of PD-1 inhibitors to PD-1 prevents the interaction between PD-L1 and PD-1, thereby disrupting the transmission of immune inhibitory signals. This allows for the restoration of the activity and function of T cells, enabling them to more effectively attack tumor cells. The use of PD-1 inhibitors enhances the immune system’s response to tumors and improves the effectiveness of tumor treatment; ③ The activity and function of T cells are restored, allowing them to more effectively attack tumor cells.

Currently, PD-1 inhibitors, such as nivolumab, pembrolizumab, and pemigatinib, have demonstrated good efficacy in treating various malignant tumors. These inhibitors bind to the PD-1 receptor, blocking the interaction between PD-1 and PD-L1. This releases the inhibition on T cells, allowing them to regain their ability to attack cancer cells. As a result, activated T cells release cytotoxins and cytokines to attack cancer cells, enhancing the immune response. Additionally, PD-1 inhibitors can promote the formation of memory T cells and enhance the immune system’s sustained ability to attack cancer cells (Khasraw et al., 2020; Liu J. et al., 2021; Yang Z. et al., 2022) (Figure 1).

Despite significant progress in cancer treatment with PD-1 inhibitors, there are still some potential limitations. Firstly, not all patients respond favorably to PD-1 inhibitor therapy. Some patients may be insensitive to this treatment due to various factors, or they may experience tumor progression even after receiving therapy (Sun JY. et al., 2020). Additionally, PD-1 inhibitors can elicit excessive activation of the immune system, resulting in immune-related adverse events such as fatigue, rash, gastrointestinal issues, and abnormal liver function (Sun L. et al., 2020). Similar to many other drugs, patients using PD-1 inhibitors may develop resistance (Sun JY. et al., 2020), wherein cancer cells that initially responded to the drug gradually lose their sensitivity over time, leading to a decline in treatment efficacy. Consequently, further research is necessary to comprehend the mechanisms of resistance and develop novel therapeutic strategies to surmount it.

PD-1 inhibitors have been widely used in the treatment of NSCLC, bringing hope to patients with this condition. Studies have shown that PD-1 inhibitors have demonstrated good efficacy in different stages of NSCLC treatment, including metastatic NSCLC. PD-1 inhibitors have been proven to significantly prolong OS and PFS when used as first-line or subsequent-line therapies. Additionally, combining PD-1 inhibitors with traditional treatment methods like chemotherapy and radiotherapy has shown synergistic effects, further improving treatment outcomes (Reck et al., 2022; Akbar et al., 2023; Lee et al., 2023; Sakamoto and Jimeno, 2023; Curkovic et al., 2024). For example, a study by Zhu et al. treated untreated clinical stage II-III and wild-type EGFR/ALK NSCLC patients with neoadjuvant toripalimab (a PD-1 inhibitor) combined with carboplatin (Zhu et al., 2022). The results showed that this combination had good safety and efficacy in stage II-III NSCLC, providing better treatment opportunities and prognosis for patients who were originally not suitable for surgery. Another study by Yang et al. (Yang X. et al., 2022) analyzed the results of lung cancer patients with brain metastases and found that PD-1/PD-L1 inhibitors significantly improved the treatment efficacy, prolonging OS and PFS compared to chemotherapy. Wankhede et al. analyzed 12 randomized controlled studies and found that compared to chemotherapy, PD-1/PD-L1 inhibitor-chemotherapy combination regimens were more likely to achieve objective response in NSCLC patients (Wankhede et al., 2023). Although PD-1 inhibitors have shown many potential advantages in the treatment of NSCLC, there are also some adverse reactions that may occur in patients. Common adverse events include skin-related reactions like rash, itching, and vitiligo, as well as systemic adverse reactions like fatigue, nausea, and muscle and joint pain (Chen J. et al., 2021; Curkovic et al., 2024). Therefore, close monitoring of patients’ adverse reactions and timely implementation of corresponding treatment measures are necessary when using PD-1 inhibitors to treat NSCLC.

In summary, PD-1 inhibitors have become an important treatment option in NSCLC as a novel immunotherapy drug. They significantly improve the prognosis of patients with metastatic NSCLC by activating the patient’s own immune system and provide new treatment opportunities for early-stage NSCLC patients. However, further research is needed to explore the optimal use of PD-1 inhibitors in NSCLC treatment and develop personalized treatment strategies for patients.

## 4 The impact of sarcopenia on the efficacy of PD-1 inhibitor treatment in NSCLC

Previous studies have indicated that body composition might be a contributing factor to the efficacy of PD-1 inhibitors in NSCLC treatment. Both fat and muscle content could influence the effectiveness of PD-1 inhibitors (Antoun et al., 2023; Roccuzzo et al., 2023). To identify relevant articles regarding the impact of sarcopenia on the efficacy of PD-1 inhibitor treatment for NSCLC, we conducted searches across multiple academic databases including PubMed, Scopus, Google Scholar, J-stage, and Web of Science. Additionally, manual searches were performed to ensure the inclusion of the most relevant studies. We excluded non-original research such as review articles, book chapters, conference abstracts, and articles unrelated to the topic of this review. We specifically selected articles that implemented clinical research designs to ensure reasonable study designs, reliable methodologies, sufficient sample sizes, and clear results and conclusions. Following the aforementioned screening process, we obtained a total of 10 relevant articles (Multani et al., 2019; Nishioka et al., 2019; Shiroyama et al., 2019; Cortellini et al., 2020; Takada et al., 2020; Rounis et al., 2021; Wang et al., 2021; Nishioka et al., 2022; Makrakis et al., 2023; Tenuta et al., 2023) (See Table 2).

**TABLE 2:** Related studies on the impact of sarcopenia on the therapeutic effect of PD-1 inhibitors in NSCLC.

References	Participants		NSCLC Stage/severity	PD-1 inhibitors	Primary study results
	n	Age (years)			
Shiroyama.et al. (Shiroyama et al., 2019)	42	(37–87)	Advanced	Nivolumab/Avelumab	Patients with sarcopenia had significantly lower PFS (2.1 months vs. 6.8 months), ORR (40.0% vs. 9.1%), and 1-year PFS rate (38.1% vs. 10.1%) compared to patients without sarcopenia
Nishioka.et al. (Nishioka et al., 2019)	38	68.7 (46–85)	Advanced	Nivolumab/Avelumab	The sarcopenia group had significantly shorter PFS compared to the non-sarcopenia group, and the RDW values were significantly higher in the sarcopenia group compared to the non-sarcopenia group
Multani.et al. (Multani et al., 2019)	100	68	IIIB/IV	-	Patients with SMI <32 cm^2^/m^2^ had significantly lower OS (median 1.41 months)
Cortellini.et al. (Cortellini et al., 2020)	100	25–88	-	-	SMI is an independent predictor of OS. There was no significant difference in median PFS and OS between patients with low SMI and those without low SMI.
Takada.et al. (Takada et al., 2020)	103	67 (36–88)	IIIB-IV/Relapse	Nivolumab/Avelumab	Low L3-SMI is an independent predictor for PFS and OS, with the low L3-SMI group having significantly lower disease control rate compared to the high L3-SMI group (49.0% vs. 73.1%)
Wang.et al. (Wang et al., 2021)	105	55 (28–77)	IV	Nivolumab/Pembrolizumab/Cetrelimab	Patients with sarcopenia had significantly lower PFS (2.67 months vs. 7.96 months) and OS (9.08 months vs. 21.84 months) compared to patients without sarcopenia. Sarcopenia was associated with the NLR.
Rounis.et al. (Rounis et al., 2021)	83	66 (39–81)	Advanced	-	Malnutrition is associated with a decreased response rate to ICIs and is an independent predictor of adverse survival outcomes
Nishioka.et al. (Nishioka et al., 2022)	74	67.5 (33–84)	Advanced	Pembrolizumab/Nivolumab/Atezolizumab	In patients with cachexia, there were no significant differences in ORR and PFS between the total adipose tissue maintenance group and the total adipose tissue reduction group
Makrakis.et al. (Makrakis et al., 2023)	52	68 (39–81)	Advanced		Low L-SMI is associated with poorer overall survival in patients
Tenuta.et al. (Tenuta et al., 2023)	47	67 (61; 74)	IV	Nivolumab/Pembrolizumab/Atezolizumab	Patients with sarcopenia have lower values of CD3-CD56^+^ NK cells and CD56dim NK cells

PFS, Progression-Free Survival; OS, overall survival; NLR, neutrophil-to-lymphocyte ratio; SMI, skeletal muscle index; L-SMI, lumbar skeletal muscle index; RDW, red cell distribution width; ICIs, Immune Checkpoint Inhibitors; ORR, overall response rate.

Rounis et al. discovered that weight loss associated with NSCLC is linked to a decreased response rate to ICIs, and weight loss independently predicts poorer survival rates (Rounis et al., 2021). This suggests that changes in body composition may influence the outcomes of patients undergoing ICI treatment. In contrast, Nishioka et al. found that in NSCLC patients with weight loss, the maintenance or reduction of total fat tissue did not significantly affect the overall objective response rate (ORR) and progression-free survival (PFS) (Nishioka et al., 2022). However, in patients without weight loss, the group with reduced total fat tissue exhibited significantly higher ORR and PFS compared to the maintenance group. Cortellini et al. and Makrakis et al. additionally revealed that muscle loss has a negative impact on NSCLC patients receiving PD-1 inhibitors, where lower muscle parameters indicate poorer OS (Cortellini et al., 2020; Makrakis et al., 2023).

Multani et al. conducted an observational study on the efficacy of PD-1 inhibitors in 100 patients with stage IIIB/IV NSCLC. They evaluated the skeletal muscle using the cross-sectional area of the lumbar muscles on CT scans (Multani et al., 2019). The results showed that patients with a Lumbar Skeletal Muscle Index <32 cm^2^/m^2^ had significantly lower OS. Stage IV is typically considered advanced or metastatic lung cancer, indicating that the tumor has spread extensively to distant organs, and patients at this stage usually have a poor prognosis. Tenutad et al. studied 47 patients with stage IV NSCLC treated with Nivolumab/Pembrolizumab/Atezolizumab and found that patients with sarcopenia had poorer OS and PFS during PD-1 inhibitor treatment (Tenuta et al., 2023). Similarly, Wang et al. observed 105 patients with stage IV NSCLC and found that patients with sarcopenia who received anti-PD-1 immunotherapy had significantly worse PFS and OS compared to non-sarcopenic patients (Wang et al., 2021). Takada et al. conducted an observational analysis on patients with stage IIIB-IV and recurrent NSCLC treated with Nivolumab/Avelumab and found that a low third lumbar muscle index (third lumbar muscle area/height^2^, cm^2^/m^2^) was an independent predictor of PFS and OS (Takada et al., 2020). Shiroyama et al. in a retrospective study of 42 patients with advanced NSCLC receiving Nivolumab/Avelumab, also demonstrated that patients without sarcopenia had better PFS, higher ORR, and higher 1-year PFS rates compared to patients with sarcopenia (Shiroyama et al., 2019). They emphasized that the impact of sarcopenia on the efficacy of PD-1 inhibitors should not be overlooked. Nishioka et al. also observed the effects of sarcopenia on the treatment outcomes of advanced NSCLC patients and found that sarcopenia had an adverse impact on the efficacy of PD-1 treatment (Nishioka et al., 2019). The sarcopenia group exhibited significantly shorter PFS than the non-sarcopenia group, even when patients had good Performance Status (PS) scores. A decrease in the Psoas Major Muscle Area (PMMA) by more than 10% significantly compromised treatment effectiveness.

Research on other malignancies has also indicated the impact of sarcopenia on PD-1 inhibitors. Kim et al. examined 185 patients with advanced gastric cancer who received Nivolumab/Pembrolizumab treatment and found that sarcopenia predicted poor prognosis, with sarcopenic patients experiencing worse OS (Kim N. et al., 2021). Kim et al. studied patients with microsatellite-stable gastric cancer and found that those with sarcopenia had significantly shorter PFS compared to non-sarcopenic individuals. Sarcopenia was independently associated with shorter PFS (Kim YY. et al., 2021). Although sarcopenic patients had shorter OS, sarcopenia was not a significant prognostic factor for OS. Young et al. observed patients with melanoma undergoing PD-1 inhibitors and found that patients with higher skeletal muscle assessment scores had better prognosis, while those with poorer assessment scores had worse PFS and OS (Young et al., 2020). This study also noted that sarcopenic obese patients had worse PFS. However, in contrast to the aforementioned findings, Hu et al. examined 156 patients with melanoma treated with pembrolizumab and found no correlation between sarcopenia and treatment response (Hu et al., 2020). Akce et al. observed 57 patients with hepatocellular carcinoma and found no correlation between sarcopenia and OS or PFS (Akce et al., 2021). Conversely, Guo et al. studied patients with hepatocellular carcinoma treated with camrelizumab and found that sarcopenic patients had shorter PFS compared to non-sarcopenic patients (Guo et al., 2022). Even after balancing various confounding factors using Propensity Score Matching (PSM), the results remained consistent. However, there was no significant difference in OS between sarcopenic and non-sarcopenic patients after PSM. A meta-analysis conducted by Takenaka et al. revealed that malignancies accompanied by sarcopenia exhibited worse PFS with ICI treatment, suggesting a negative correlation between sarcopenia and ICI efficacy (Takenaka et al., 2021).

In conclusion, sarcopenia could potentially impact the effectiveness of PD-1 inhibitors and have adverse effects on the prognosis of NSCLC patients. Although some studies on other malignancies have reported contradictory results, this might be attributed to small sample sizes or other factors. Overall, sarcopenia may have a negative impact on the effectiveness of PD-1 inhibitors in treating NSCLC; however, further research is required to confirm this association.

## 5 Discussion

### 5.1 Possible mechanisms of sarcopenia on the impact of PD-1 inhibitors

**FIGURE 2:**
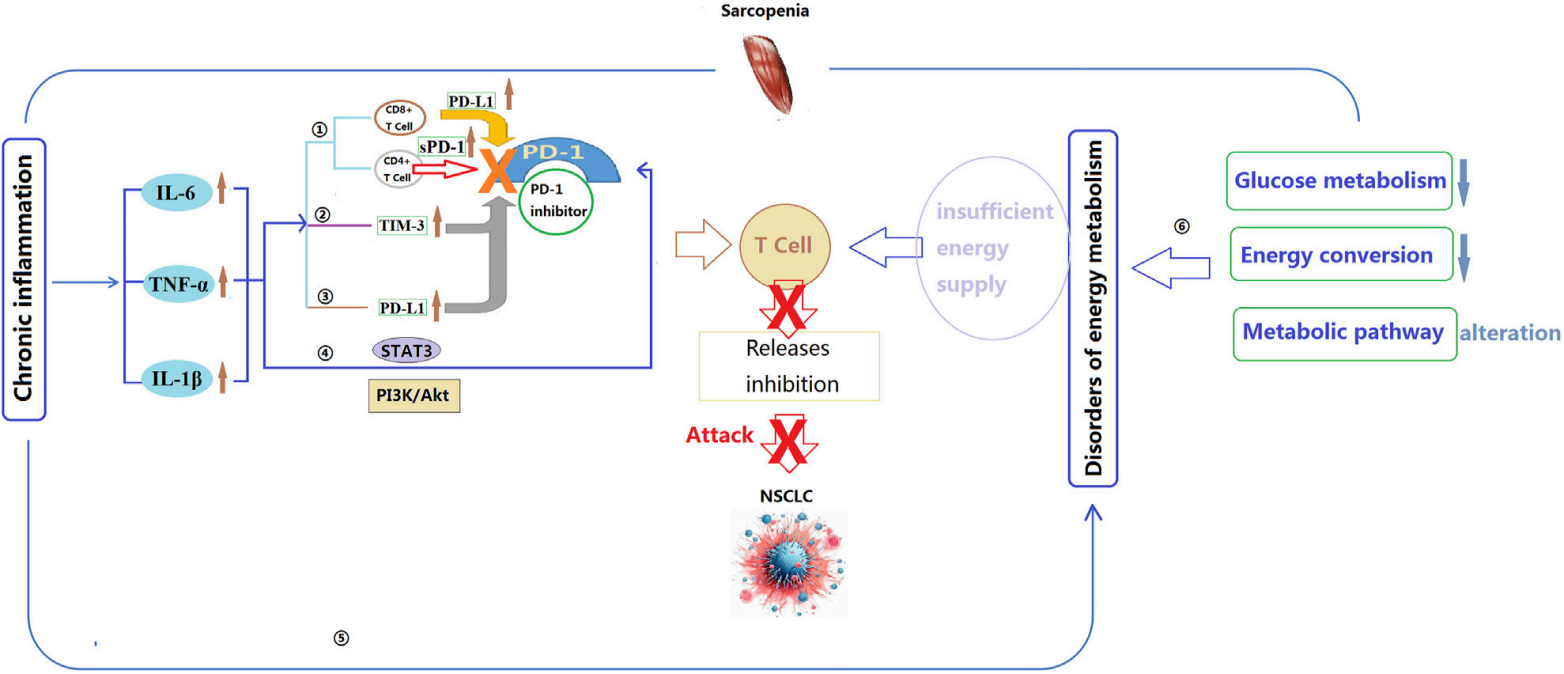
Potential mechanisms by which sarcopenia affects the effectiveness of PD-1 inhibitors. IL-6: Interleukin-6; IL-1β: Interleukin-1 beta; TNF-α: Tumor necrosis factor alpha; STAT3: Signal Transducer and Activator of Transcription 3; TIM-3: T-cell immunoglobulin and mucin domain-containing protein 3; PI3K: Phosphoinositide 3-kinase; Akt: Protein kinase B; PD-1: Programmed cell death protein 1; PD-L1: Programmed death-ligand 1. ①Inflammatory cytokines regulate the activity of CD4^+^ and CD8^+^ cells, thereby influencing the efficacy of PD-1 inhibitors; ②TNF-α can increase the expression of TIM-3, further attenuating immune response and the efficacy of PD-1 inhibitors; ③IL-6, IL-1β, and TNF-α directly interact with PD-L1, leading to an increase in its expression, potentially reducing the binding capacity between PD-1 inhibitors and PD-L1, thereby weakening the effectiveness of PD-1 inhibitors; ④Inflammatory cytokines may modulate the efficacy of PD-1 inhibitors by influencing the activity of the STAT3 and PI3K/Akt signaling pathways; ⑤Chronic inflammation may impact the efficacy of PD-1 inhibitors by modulating cellular energy metabolism and mitochondrial function; ⑥The energy metabolism abnormalities caused by sarcopenia may reduce the responsiveness of T cells and the immune evasion of cancer cells, as well as impact cellular mitochondrial function and oxidative stress levels, thereby diminishing the efficacy of PD-1 inhibitors.

Limited reports have specifically analyzed the mechanisms underlying the impact of sarcopenia on PD-1 inhibitors. However, factors such as immune dysregulation, inflammatory response, muscle metabolism, and energy balance may contribute to the influence of sarcopenia on the efficacy of PD-1 inhibitors (Figure 2).

#### 5.1.1 Chronic inflammation and immune cells suppression

A study by Tenuta et al. demonstrated that patients with non-small cell lung cancer (NSCLC) accompanied by sarcopenia have lower levels of CD3-CD56^+^ NK cells and CD56dim natural killer (NK) cells (Tenuta et al., 2023). These findings suggest a potential association between sarcopenia and alterations in immune function among NSCLC patients. CD3-CD56^+^ NK cells and CD56dim NK cells are subgroups of NK cells that play a crucial role in immune response and anti-tumor reactions. Reduced levels of NK cells may lead to a decreased immune response against cancer, thereby increasing the susceptibility and disease progression risk in NSCLC patients. Wang et al. also found a certain correlation between sarcopenia and the neutrophil-lymphocyte ratio (NLR), which is used to assess inflammation and immune status (Wang et al., 2021). An increase in NLR is typically associated with changes in inflammation response and immune function. One possible factor contributing to these changes is the elevation of pro-inflammatory cytokines associated with sarcopenia. Changes in pro-inflammatory cytokines may impact the function of immune cells. These pro-inflammatory cytokines are involved in regulating immune responses and inflammatory processes. For example, elevated levels of IL-6 can trigger inflammatory responses, attract and activate immune cells, and affect various immune cell types, including T cells, B cells, NK cells, and monocytes. Ultimately, this may result in increased proliferation and activation of T cells, promotion of B cell differentiation and antibody production, as well as regulation of NK cell activity (Yadav et al., 2009; Takenaka et al., 2021). Therefore, chronic inflammation may play a significant role in the relationship between sarcopenia and PD-1 inhibitors.

Chronic inflammation is considered an adaptive response to tissue dysfunction or imbalances in homeostasis and is believed to underlie various physiological and pathological processes. Sarcopenia has been associated with chronic inflammation, as it is accompanied by higher levels of inflammatory markers such as IL-6. Elevated levels of IL-6, IL-1β, TNF-α, and C-reactive protein (CRP) have been implicated in the loss of muscle mass and strength in sarcopenia (Icard et al., 2018; Ushitani et al., 2022). Elevated levels of IL-6, IL-1β, TNF-α, and CRP have been identified as potential contributors to the unfavorable outcomes of sarcopenia in PD-1 inhibitor treatment. Previous studies have demonstrated that sarcopenic patients exhibit higher levels of TNF-α, IL-1β, IL-6, and CRP (Lee et al., 2007; Koshikawa et al., 2020; Pan et al., 2021; Shokri-Mashhadi et al., 2021; Chang et al., 2023). IL-6 is primarily produced by monocytes-macrophages, T helper 2 cells (TH2), and other cells, and it plays a role in the differentiation of immune cells. It acts as an endogenous pyrogen involved in inflammatory responses. Due to signaling pathway factors, IL-6 exhibits both pro-inflammatory and anti-inflammatory activities. Pro-inflammatory IL-6 can interfere with skeletal muscle protein synthesis and directly contribute to skeletal muscle protein degradation, resulting in a decrease in skeletal muscle mass. As a classic inflammatory factor, TNF-α also promotes muscle breakdown. Research has found that TNF-α can affect protein expression by inhibiting lipopolysaccharide activity and can exacerbate protein degradation in muscles, promoting muscle breakdown. IL-1β is an intracellular cytokine belonging to the IL-1 family and plays a key role in coordinating innate and adaptive immune responses. Currently, there is relatively limited research on IL-1β and sarcopenia, but studies have shown its potential correlation with grip strength (Rose-John, 2012; Nishikawa et al., 2021a; Shokri-Mashhadi et al., 2021; Chang et al., 2023; Dupont et al., 2023).

**FIGURE 3:**
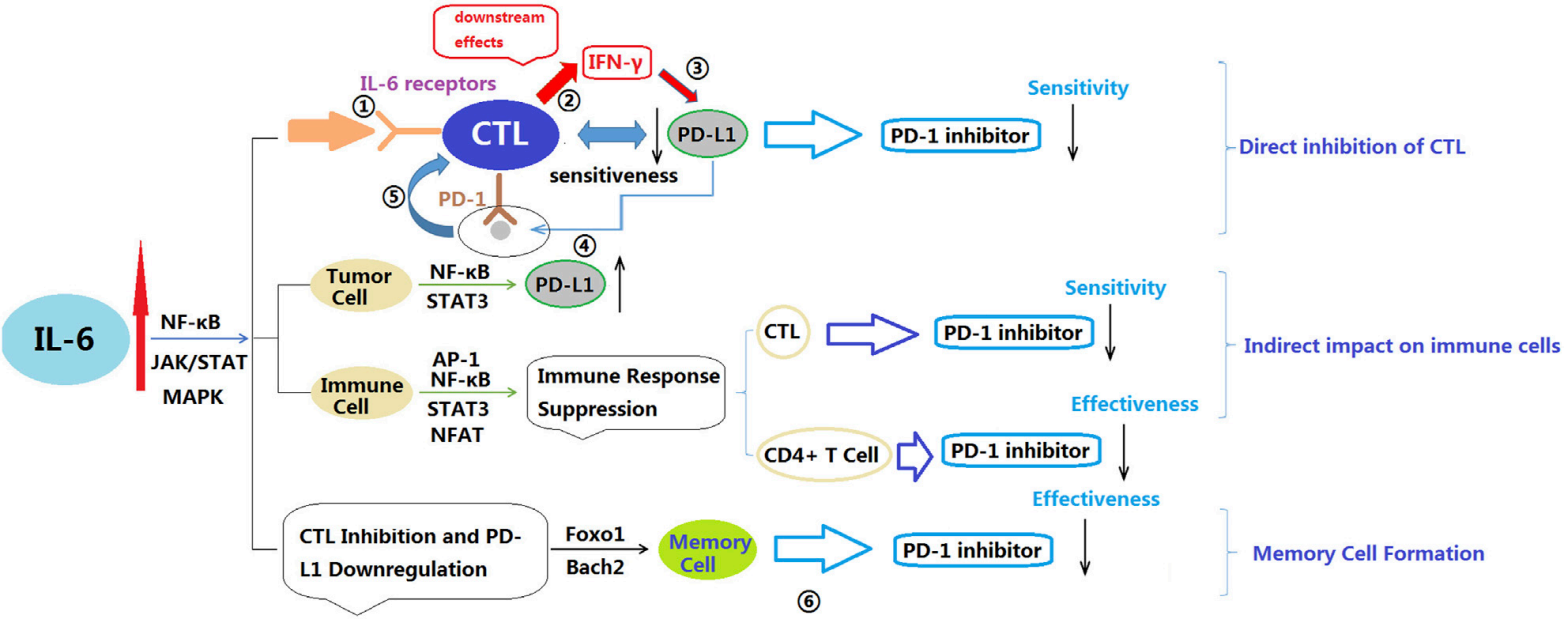
Potential mechanisms by which IL-6 affects PD-1 inhibitors. IL-6: Interleukin-6; NF-κB: Nuclear factor kappa-light-chain-enhancer of activated B cells; JAK: Janus kinase; STAT: Signal transducer and activator of transcription; MAPK: Mitogen-activated protein kinase; FOXO: Forkhead box O; INF-γ: Interferon gamma; CTL: Cytotoxic T lymphocyte. ①IL-6 binds to IL-6 receptors on CTL; ②IL-6 signaling is activated, leading to downstream effects. Downstream effects of IL-6 signaling include suppression of CTL effector gene expression, such as interferon-gamma (IFN-γ); ③Reduced expression of effector genes decreases CTL sensitivity to PD-L1; ④PD-L1 on tumor cells binds to PD-1 on CTL; ⑤The interaction between PD-L1 and PD-1 inhibits CTL activity, Due to reduced sensitivity to PD-L1, CTL cannot effectively overcome the inhibitory effect; ⑥IL-6 inhibits memory cell development and function through its interaction with FOXO proteins, potentially reducing the effectiveness of PD-1 inhibitors.

Bommarito et al. found that the inflammatory cytokines TNF-α, IL-6, and IL-1β act as negative regulators of PD-1-mediated T cell suppression *in vitro* (Bommarito et al., 2017). They can reduce the activity or expression levels of PD-1, diminishing the effectiveness of PD-1 binding to its ligands. This implies that T cells may not receive sufficient signals to release inhibition, limiting their ability to eliminate tumor cells. Additionally, the expression level of PD-1 also partially determines the response to PD-1 inhibitors. If the expression level of PD-1 on the surface of tumor cells is low, PD-1 inhibitors may not effectively bind to PD-1 on tumor cells, limiting their therapeutic effect. Furthermore, TNF-α and IL-6 can induce CD4^+^ T cells to secrete soluble PD-1 (sPD-1), which interferes with effective PD-1 binding and further reduces the efficacy of PD-1 inhibitors. Keegan et al. conducted an observational study on NSCLC patients receiving anti-PD-1 treatment and measured 11 plasma cytokines related to immunotherapy response (Keegan et al., 2020). The results showed that decreasing levels of IL-6 were associated with improved PFS. Additionally, this study also observed a correlation between changes in IL-6 and changes in CRP levels. CRP is a protein associated with inflammation and tissue damage and is typically elevated during an inflammatory response. Therefore, the changes in IL-6 and CRP may indicate their association with the inflammatory process. The authors suggested that the decrease in plasma IL-6 and CRP levels was associated with improved prognosis in NSCLC patients receiving anti-PD-1 treatment. In the study of Park et al. involving 125 NSCLC patients receiving PD-1/PD-L1 inhibitor treatment, found that the low IL-6 group (<13.1 pg/mL) had significantly higher ORR and disease control rate (DCR) compared to the high IL-6 group (Kang et al., 2020). Moreover, the low IL-6 group had significantly longer PFS and OS compared to the high IL-6 group. Serum IL-6 levels may serve as a potential biomarker for predicting the efficacy and survival benefits of PD-1/PD-L1 inhibitors in NSCLC. Koeppen et al. conducted observations on the treatment of advanced cancer patients with Atezolizumab (Koeppen et al., 2023). The results showed a correlation between IL-6 and the poor efficacy of Atezolizumab in advanced cancer patients. The IL-6 signal within CD8^+^ T cells may directly inhibit the functionality of cytotoxic T lymphocytes (CTLs), reducing their sensitivity to PD-L1 and resulting in a decreased effectiveness of PD-L1 inhibitors. And Li et al. found in their study that blocking IL-6 promoted the accumulation of CD8^+^ T cells and led to high expression of PD-L1 in colorectal cancer, enhancing the sensitivity of animals to PD-1 therapy (Li et al., 2022a). IL-6 affects the cytotoxic effects and intracellular signaling of CD8^+^ T cells, further influencing the efficacy of PD-L1 inhibitors. By activating the STAT3 pathway, IL-6 inhibits the differentiation of CD8^+^ T cells into cytotoxic effector cells, leading to a tendency for CD8^+^ T cells to remain in an immature or memory-like state rather than developing into cells with cytotoxic effects. On the other hand, the intracellular IL-6 signaling in CD8^+^ T cells limits the response to PD-L1 inhibitors. These mechanisms may collectively reduce the efficacy of PD-L1 inhibitors (Tezera et al., 2020; Li et al., 2022a; Koeppen et al., 2023). In summary, the mechanisms by which IL-6 affects PD-1 inhibitors can be summarized as follows: 1) IL-6 directly suppresses the expression of effector genes in CTLs, reducing their cytotoxicity and effector differentiation capability, thus diminishing the efficacy of PD-1. 2) Indirectly, IL-6 impacts immune cell function by supporting tumor cell survival, inhibiting Th1 responses, promoting the generation of immunosuppressive myeloid cells, and disrupting conventional dendritic cells (cDCs), thereby reducing the efficacy of PD-1 inhibitors. 3) IL-6 promotes the expression of factors involved in memory cell formation, such as Foxo1, IL-10, and Bach2, which may result in CTLs favoring a naïve or memory-like state rather than differentiating into effector cells, ultimately limiting the efficacy of PD-1 inhibitors. It is important to note that the mechanisms of IL-6’s impact can be complex, involving various cell types and signaling pathways. Additionally, the influence of IL-6 on PD-1 inhibitors may vary depending on the specific tumor type and individual differences, necessitating further research in this area (Bommarito et al., 2017; Kang et al., 2020; Keegan et al., 2020; Tezera et al., 2020; Li et al., 2022a; Koeppen et al., 2023) (Figure 3)

PD-1 inhibitors are designed to alleviate the suppressive impact of PD-1 signaling on immune cells, thereby boosting the immune system’s ability to clear *mycobacterium tuberculosis* infections. Bommarito et al. discovered that TNF-α promotes the generation of sPD-1 in CD4^+^ T cells. sPD-1, though structurally similar to membrane-bound PD-1 (mPD-1), exists in a soluble form in bodily fluids (Bommarito et al., 2017). Elevated sPD-1 production may disrupt the interaction between PD-1 inhibitors and mPD-1 or PD-L1 on cell membranes, potentially compromising PD-1 inhibitor efficacy. Tezera et al. conducted a study using a three-dimensional cell culture model of human tuberculosis infection to investigate the interaction between TNF-α and PD-1 inhibitors (Tezera et al., 2020). The results demonstrated the significant role of TNF-α in tuberculosis, playing a crucial role in an effective host immune response. TNF-α can activate host immune cells and induce cytotoxic activity, production of inflammatory cytokines, to combat *mycobacterium tuberculosis* infection. When using PD-1 inhibitors for treatment, it may enhance the production of TNF-α and host immune response, leading to excessive TNF-α production. Excessive TNF-α can cause immune-mediated tissue damage and inflammatory reactions, exacerbating the severity of tuberculosis and resulting in a reduced efficacy of PD-1 inhibitors. A mathematical modeling study by Lai et al. on the combined treatment of anti-PD-1 and anti-TNF-α further revealed that TNF-α can promote the production of PD-L1, diminishing the effectiveness of anti-PD-1 drugs (Lai et al., 2020). TNF-α can also activate the expression of T-cell immunoglobulin mucin-3 (TIM-3), allowing cancer cell-expressed Galectin-3 (Gal-3) to block T cells by binding to TIM-3 on the T cell membrane. Additionally, the study showed that the combination of anti-TNF-α therapy significantly reduces resistance to PD-1 inhibitors. In summary, TNF-α may impact the function and interactions of immune cells through various mechanisms, including promoting the production of sPD-1, increasing the expression of PD-L1, activating TIM-3, and inducing immune-mediated tissue damage and inflammatory responses. These mechanisms could potentially diminish the efficacy of PD-1 inhibitors. However, further research is needed to deepen our understanding of the specific roles and underlying mechanisms of TNF-α in PD-1 inhibitor therapy.

Multiple studies on various malignancies have found a positive correlation between IL-1β levels and PD-L1 expression (Bar et al., 2020; Takahashi R. et al., 2021; Lu et al., 2021; Castillo et al., 2023). Aggen et al. conducted a study using a renal carcinoma (RENCA) cell line model of renal cancer and observed that the combination of anti-IL-1β and PD-1 inhibitors effectively enhanced anti-tumor activity. This enhancement was associated with a reduction in immunosuppressive myeloid-derived suppressor cells (MDSCs) and an increase in M1-type tumor-associated macrophages (TAMs) (Aggen et al., 2021). MDSCs can inhibit the activity and function of T cells through various mechanisms, thereby suppressing anti-tumor immune responses. TAMs, on the other hand, are one subtype of macrophages found in tumor tissues. M1-type TAMs are considered to possess anti-tumor activity and can support anti-tumor immune responses and inflammatory reactions through the production of immunostimulatory cytokines and anti-angiogenic factors.

In conclusion, elevated levels of inflammatory markers such as IL-6, IL-1β, TNF-α, and CRP in sarcopenic patients have the potential to influence the efficacy of PD-1 inhibitors. These markers may suppress the activity or expression levels of PD-1 and compromise the binding efficiency between PD-1 and its ligands, thereby limiting the release of inhibitory signals to T cells and diminishing their capacity to eliminate tumor cells. Modulating the levels of inflammatory cytokines could potentially enhance the effectiveness of PD-1 inhibitors. Nevertheless, further research is necessary to fully understand the underlying mechanisms and explore strategies for modulating inflammatory cytokines to improve the therapeutic efficacy of PD-1 inhibitors.

#### 5.1.2 Disorders of energy metabolism

During the body’s fight against infections and maintenance of survival, immune cells undergo continuous changes and adjustments to effectively carry out their defense, surveillance, and homeostasis functions. These changes often require corresponding alterations in the metabolic system to provide the necessary energy and nutrients. Conversely, changes in the metabolic system can also influence the behavior and response of immune cells, thereby tightly linking the immune system with basal metabolic tissues and participating in various life processes. Skeletal muscle, in addition to being a motor organ, plays a crucial role in insulin-induced glucose metabolism. It is involved in the storage, distribution, consumption, and conversion of energy, and is closely connected to overall metabolism and bodily functions. The decrease in muscle mass associated with sarcopenia leads to reduced energy expenditure, which can result in insulin resistance and hinder the effective utilization of glucose and regulation of blood glucose levels (Nishikawa et al., 2021b). Therefore, sarcopenia inevitably impacts overall energy metabolism in the body, and the occurrence of sarcopenia will inevitably have an impact on energy metabolism in the body. Lira et al. conducted a study on metabolic differences between upper and lower limbs after high-intensity intermittent exercise, and the results showed significant differences in metabolic levels between the upper and lower limbs of the participants (Lira et al., 2015). The lower limbs with richer muscles had higher glucose and lactate metabolism rates. Shoemaker et al. studied elderly sarcopenic patients and found differences in skeletal muscle energy metabolism and substrate utilization between sarcopenic and non-sarcopenic participants, with sarcopenic participants showing metabolic inflexibility (Shoemaker et al., 2022). Almeida et al. studied by meta-analysis revealed significantly inadequate intake of energy and nutrition substances with antioxidant potential in sarcopenic patients (Almeida et al., 2023).

Both cancer cells and T cells require energy metabolism to sustain their activities. Cancer cells primarily rely on glycolysis to generate energy, even in the presence of oxygen, a phenomenon known as the Warburg effect. Additionally, cancer cells can utilize protein and fatty acid metabolism to meet their energy and biosynthetic demands for proliferation. T cells, on the other hand, use both glycolysis and oxidative phosphorylation pathways to generate energy during activation. Naive and memory T cells rely more on oxidative phosphorylation, while effector T cells depend more on glycolysis. These distinct metabolic strategies of T cell subsets offer potential targets for metabolic interventions in tumor immunotherapy (Kumar and Chamoto, 2021). PD-1 blockade therapy, by blocking the interaction between PD-1 and its ligand PD-L1, can restore the immune response of T cells against cancer cells. However, if cancer cells can evade immune responses through specific energy metabolism pathways, it may reduce the efficacy of PD-1 blockade therapy. For example, cancer cells can affect immune regulation by releasing metabolites and influencing the expression of immune molecules, limiting the availability of nutrients, and causing microenvironmental acidosis, thereby impairing immune cell function. In addition, if T cells face energy supply insufficiency and metabolic restrictions in the tumor microenvironment, it may reduce the efficacy of PD-1 blockade therapy. Therefore, intervention in these metabolic pathways can help overcome resistance to PD-L1/PD-1 inhibitors, enhance immune cell responses, and improve treatment outcomes (Kumar and Chamoto, 2021; Xia et al., 2021; Laubach et al., 2023).

As previously mentioned, sarcopenia can induce an inflammatory state, which can potentially alter the energy metabolism of immune cells and impact their response to PD-1 inhibitors. Firstly, in an inflammatory state, cells require increased energy to meet the demands of the inflammatory response, leading to a shift towards glycolysis and fatty acid metabolism to meet their energy requirements. This change in energy metabolism may affect the functionality and responsiveness of immune cells. Secondly, the inflammatory state can influence mitochondrial function in cells. Mitochondria are the primary sites for energy production, and the presence of inflammation may lead to mitochondrial dysfunction, resulting in reduced energy production and potentially decreasing the activity and responsiveness of immune cells. Additionally, the inflammatory state can increase oxidative stress and the production of reactive oxygen species, which can damage cells and impact normal functionality (Gaber et al., 2017; Riley and Tait, 2020; Russo et al., 2021). Therefore, the inflammatory state’s influence on the energy metabolism, mitochondrial function, and oxidative stress in immune cells may affect their sensitivity to PD-1 inhibitors and subsequently impact the medication’s efficacy. However, further research is required to better comprehend the mechanisms and implications of the interaction between immune cells and PD-1 inhibitors in the context of an inflammatory state.

In conclusion, sarcopenia can potentially impact the energy metabolism of both T cells and cancer cells. Insufficient energy supply for T cells may reduce their function and responsiveness, leading to a diminished immune response against tumor cells, which may result in decreased efficacy of PD-1 inhibitors. For cancer cells, sarcopenia may lead to energy supply insufficiency and limit the availability of nutrients, enabling cancer cells to evade immune responses and weaken the effectiveness of PD-1 inhibitors. Future research can further investigate the mechanisms of sarcopenia’s impact on the efficacy of PD-1 inhibitors through clinical observations and experimental studies. For example, comparing the differences in energy metabolism pathways, metabolic products, and expression levels of metabolic enzymes between animal models with sarcopenia and without sarcopenia receiving PD-1 treatment; comparing immune cell functions and other indicators. These methods can provide guidance and new intervention strategies for immune therapy in sarcopenic patients.

### 5.2 Potential strategies to overcome the effect of sarcopenia on the efficacy of PD-1 inhibitors

To counteract the impact of sarcopenia on the efficacy of PD-1 inhibitors, it is essential to implement proactive strategies for sarcopenia prevention and treatment in cancer patients. The following aspects can be considered.

#### 5.2.1 Exercise and rehabilitation training

Exercise has been shown to have positive effects on sarcopenia, particularly in the elderly. It can help maintain skeletal muscle mass, increase muscle strength, and provide anti-inflammatory and antioxidant benefits. In the absence of effective pharmacological treatments for sarcopenia, exercise is considered an ideal approach for prevention and treatment. Resistance training has been found to be an effective strategy for slowing down sarcopenia. It stimulates muscle protein synthesis, improves muscle mass, strength, balance, and endurance (Chen N. et al., 2021). The signaling pathways involving mTOR and FoxO transcription factors play important roles in the development of sarcopenia and muscle atrophy. Resistance exercise can activate the Akt/mTOR signaling system and inhibit the expression of FoxO1/MuRF1 and FoxO3a, thereby promoting positive protein metabolism and inhibiting muscle protein breakdown (Sugimoto, 2012; Yin et al., 2020; Zeng et al., 2020; Hsu et al., 2023).

Aerobic exercise, characterized by rhythmic and repetitive movements, mainly utilizes aerobic metabolism and oxygen to benefit cardiovascular health, body composition, and the heart and respiratory system. It can reduce the expression of catabolic proteins and increase protein synthesis, promoting muscle growth (Park, 2019). Aerobic exercise training also activates mitochondrial biogenesis, improves mitochondrial function and metabolic capacity, reduces oxidative stress and catabolic pathways, decreases muscle protein breakdown, and provides sufficient energy for protein synthesis (Konopka and Harber, 2014). Combining aerobic exercise and resistance exercise can enhance muscle function and cardiopulmonary function, making it an ideal intervention for sarcopenia. Aerobic exercise helps reduce oxidative stress and apoptosis caused by mitochondrial dysfunction, while resistance exercise improves muscle mass and function. High or moderate certainty evidence suggests that the combination of resistance exercise and aerobic exercise is the most effective intervention for improving the quality of life in sarcopenic elderly individuals. Comprehensive exercise programs have been shown to improve body composition and effectively reduce sarcopenia-related risk factors (Yoo et al., 2018; Shen et al., 2023).

Current research suggests that exercise has the potential to improve health outcomes in cancer survivors by increasing muscle mass and reducing sarcopenia. Additionally, for lung cancer patients, exercise can modulate the immune system, effectively reducing levels of inflammatory markers and increasing the production of anti-inflammatory cytokines, which can help alleviate systemic inflammation caused by lung cancer and reduce muscle wasting. Exercise also improves muscle metabolism and function, increasing the rate of muscle protein synthesis and reducing muscle breakdown (Cao et al., 2022; Cortiula et al., 2022). There is evidence to suggest that aerobic exercise training has significant benefits for patients with NSCLC after thoracoscopic surgery (Do et al., 2023). Rutkowska et al. found that NSCLC patients who underwent exercise training demonstrated significant improvements in the 6-min walk test, leading to enhanced physical fitness (Rutkowska et al., 2019). The exercise group also exhibited improvements in lung function parameters, highlighting the benefits of exercise training on lung function. Furthermore, exercise training positively impacted dynamic balance and coordination, reducing the risk of falls. Additionally, respiratory distress was alleviated in the exercise group, suggesting that exercise training may reduce fatigue symptoms and enhance exercise endurance. Hwang et al. demonstrated that exercise training can improve exercise capacity and peak oxygen consumption in NSCLC patients receiving targeted therapy, which is an important predictor of long-term prognosis (Hwang et al., 2012). Preoperative exercise training has also been shown to improve exercise capacity, shorten hospital stay, and reduce postoperative complications in NSCLC patients. Furthermore, Rosero et al. conducted a 10-week exercise intervention program for NSCLC patients showed significant improvements in muscle performance, strength, walking speed, standing ability, and overall physical function in the intervention group (Rosero et al., 2020). However, there is still a lack of research on exercise interventions for muscle in NSCLC patients, and more studies are needed to provide evidence for developing appropriate exercise plans for patients. In conclusion, appropriate exercise plans can be tailored to individual patients’ physical condition, treatment regimen, and abilities, combined with rehabilitation training, to help restore muscle function and strength. Further research is needed to better understand the specific benefits of exercise for NSCLC patients and to develop effective exercise interventions.

#### 5.2.2 Nutrition support

Nutritional supplementation has been shown to be effective in delaying and controlling sarcopenia. Combining supplementation with exercise can potentially yield even better results. It is crucial to ensure that patients consume sufficient protein to support muscle repair and growth. Oral nutritional supplements were initially recommended to address inadequate dietary intake and potential malnutrition. Sarcopenic and malnourished patients may struggle to obtain enough protein and other nutrients through diet alone. In such cases, oral nutritional supplements are highly suitable for providing high-quality nutrition (Cawood et al., 2012; Cramer et al., 2016). A prospective study conducted by Cramer et al. on sarcopenic patients for 24 weeks showed that using high-protein oral nutritional supplements can help sarcopenic patients maintain and rebuild muscle mass and strength. In mild sarcopenic patients, compared to the control group’s intake (20 g protein; 499 IU vitamin D3; taken twice daily between meals), the group receiving standard intake (14 g protein, 147 IU vitamin D3; taken twice daily between meals) had more favorable effects on leg muscle strength and mass improvement (Caccialanza et al., 2022). NSCLC patients often experience various degrees of nutritional impairment, leading to chronic inflammation, wasting, and loss of appetite. Anti-cancer treatments themselves, such as radiation therapy, chemotherapy, and surgery, can also cause severe malnutrition in patients. Abnormal nutritional status is correlated with poorer prognosis and the need for more frequent interruptions/delays in cancer treatment (Cramer et al., 2016). A study by Detopoulou et al. on 82 male NSCLC patients showed a potential relationship between a diet rich in potatoes and animal protein and patient prognosis, indicating the crucial role of protein in cancer management and preventing muscle wasting (Detopoulou et al., 2022). However, there is currently insufficient research on the specific protein intake for cancer-related sarcopenic patients. More clinical data is needed to support and establish appropriate nutritional supplementation standards (Ford et al., 2021; Pimentel et al., 2021; Capitão et al., 2022).

One of the characteristics of cachexia in malignant tumors is the significant loss of muscle mass, which is believed to be influenced by chronic systemic inflammation and oxidative stress. Research has shown that serum carnitine levels are lower in cachexia patients compared to healthy controls, with a difference of 8.20 μmol/L (*p* < 0.000) for free carnitine and 2.60 μmol/L (*p* = 0.029) for short-chain acylcarnitine. These changes are considered to play an important role in the development of cachexia. Studies have been conducted to supplement carnitine in such patients, and the results have been quite promising (Vinci et al., 2005; Silvério et al., 2011). Levocarnitine is a form of carnitine and a conditionally essential amino acid-like molecule that is primarily found in skeletal muscles. It plays a central role in fatty acid metabolism and exhibits significant antioxidant and anti-inflammatory properties. Animal studies have shown that supplementation with a 28-day dose (0.1 g) of levocarnitine can promote muscle recovery (Silvério et al., 2011; Evans et al., 2017). Evans et al. conducted a study on middle-aged and elderly individuals and found that supplementation with a combination formula containing 1,500 mg/day of levocarnitine (along with leucine and creatine) for 8 weeks increased muscle mass by 1.0 kg, significantly improved lower limb lean mass, calf strength, and non-trunk lean mass (Evans et al., 2017). The combination of levocarnitine and leucine in the formula can stimulate muscle protein synthesis when combined with whey protein isolate, while creatine can increase muscle mass and strength. The addition of levocarnitine can increase the bioavailability of branched-chain amino acids and reduce protein degradation, possibly due to improved protein metabolism through the mTOR pathway. Kraft et al. conducted a study on pancreatic cancer patients and found that supplementing with levocarnitine for 3 months significantly increased body weight, improved body composition, and improved quality of life in advanced pancreatic cancer patients. Levocarnitine may have potential therapeutic effects on cancer cachexia (Kraft et al., 2012).

Future research can investigate more precise nutritional intake strategies, encompassing diverse protein types and sources, along with the synergistic utilization of nutritional supplementation and exercise training. Personalized nutritional interventions based on individual characteristics and metabolic status hold promise as a research direction. Through further clinical research and long-term follow-up observations, a better understanding of the effects of different intervention strategies on sarcopenia can be gained, leading to the development of more specific nutritional treatment plans for NSCLC patients receiving PD-1 inhibitors.

#### 5.2.3 Drug intervention

Drug intervention is another avenue worth considering. Anamorelin is a medication that has been investigated for its potential in ameliorating cancer cachexia. Presently, Anamorelin has received approval in certain countries for treating cancer cachexia in select cancer patient populations. Anamorelin may improve cachexia through multiple mechanisms. As a Ghrelin receptor agonist, it can increase appetite and promote protein synthesis and muscle mass. Additionally, Anamorelin may also alleviate muscle wasting caused by cachexia by inhibiting the signaling of cytokines such as Myostatin, Activin-A, and TGF-β, which are considered important negative regulators of skeletal muscle mass. Therefore, Anamorelin may increase muscle mass and reduce muscle wasting by inhibiting the activity of these cytokines (Wakabayashi et al., 2021; Hanada et al., 2022; Takayama et al., 2023). However, clinical observations conducted by Dawson-Hughes et al. showed that Anamorelin did not significantly change muscle mass but may improve lower limb strength (Dawson-Hughes et al., 2024). While Anamorelin exhibits promise in ameliorating cancer cachexia, further research and exploration are necessary to comprehensively understand its precise mechanisms of action and its effects on sarcopenia. Additionally, the efficacy and safety of Anamorelin require further evaluation to ascertain its optimal utilization in clinical practice.

#### 5.2.4 Management of side effects and drug interactions

Throughout the course of cancer treatment, side effects like nausea, vomiting, fatigue, and loss of appetite can adversely affect patients’ nutritional intake. Therefore, healthcare professionals provide appropriate nutritional management, preventive education, and other measures as necessary to prevent iatrogenic sarcopenia (Nagano et al., 2019). Some treatment drugs can also cause muscle loss, such as glucocorticoids, and PD-1 inhibitors can stimulate the gastrointestinal tract, thereby interfering with nutrient intake (Klein, 2015; Cheng et al., 2023). Therefore, addressing iatrogenic and drug-induced sarcopenia is essential for patients. In combination with the aforementioned nutritional therapy, healthcare professionals can develop personalized nutrition plans, provide appropriate nutritional support based on the specific circumstances of the patient, and suggest the adoption of frequent small meals to avoid prolonged fasting, increase the frequency of eating, relieve the burden on the digestive system, alleviate treatment side effects, maintain metabolic stability and adequate nutrition (Barry, 2018). To address gastrointestinal reactions during PD-1 inhibitor treatment, healthcare professionals should closely monitor patients’ condition, evaluate the impact on their nutritional intake, implement suitable dietary adjustments and medication support as needed, and collaborate with gastroenterology specialists to prevent additional muscle loss.

#### 5.2.5 Psychological support and rehabilitation psychology

Cancer patients often face emotional and psychological stress, accompanied by chronic inflammation and immune system dysfunction. These factors can eventually lead to the occurrence of depression, and depression has a more severe impact on lung cancer patients compared to other types of cancer. Depression has been identified as a prognostic factor for the survival rate of NSCLC (Andersen et al., 2023). Li et al. analyzed NSCLC patients with depression and found that the group with depression had a lower PFS compared to the group without depression (Li et al., 2022b). There were statistically significant differences in ORR and DCR between the two groups, indicating that depression can affect the efficacy of chemotherapy combined with immunotherapy. Sarcopenic patients, like cancer patients, face elevated inflammatory factors, immune dysfunction, and limited physical activity, and therefore, also face depression issues. Szlejf et al. conducted a study on sarcopenia and depression among 5,927 participants aged 55 and above, and the results showed a correlation between depression and sarcopenia as well as low muscle strength (Szlejf et al., 2019). Chang et al. analyzed the relationship between sarcopenia and depression in 10 studies on myopenia, and the results also showed an independent correlation between sarcopenia and depression (Chang et al., 2017). In conclusion, NSCLC patients who develop sarcopenia may also experience depression. Depression not only affects the prognosis of NSCLC patients but also influences the effectiveness of PD-1 inhibitors. Cohen et al. observed 62 patients with advanced cancer receiving ICIs treatment and found that psychological issues can affect treatment outcomes through pro-inflammatory cytokines and Soluble cytotoxic T-lymphocyte-associated antigen 4 (sCTLA-4) (Cohen et al., 2023).

In summary, providing psychological support and rehabilitation psychology services to help patients cope with emotional and psychological distress and improve their motivation and self-management abilities is crucial. Firstly, it is important to provide patients with emotional support, helping them understand and cope with the emotional reactions they may experience, such as anxiety, fear, and depression. By offering emotional support, patients can better face the challenges of cancer and seek positive coping strategies. Secondly, assisting patients in adjusting their cognition and attitude towards cancer and helping them develop a positive mindset is essential. Providing effective coping strategies is also necessary to help patients navigate the various challenges during cancer treatment. This may include techniques and tools for managing treatment side effects, pain management, and adapting to lifestyle changes. Lastly, using the principles of rehabilitation psychology, helping patients improve their self-management abilities, including skills for diet, exercise, sleep, and stress management, contributes to enhancing their quality of life and overall rehabilitation outcomes.

#### 5.2.6 Regular monitoring and assessment

Regular evaluation of muscle mass and function is crucial for patients, and monitoring their muscle health is an essential measure. In addition to routine instrumental methods such as CT, MRI, DXA, and BIA for skeletal muscle examination, grip strength, gait speed, SARC-F, SARC-F combined with calf circumference (SARC-CalF), and grip strength testing can also be used for convenient self-testing by patients and their families. Grip strength threshold: Men: <26 kg, Women: <18 kg; Gait speed threshold: ≤0.8 m/s; SARC-F includes: 1) Lifting/carrying a 10-pound weight; 2) Walking across a room; 3) Getting up from a bed or chair; 4) Climbing 10 flights of stairs; 5) Number of falls in the past year, with three scoring options (easy: 0 points; moderately difficult: 1 point; difficult/unable to complete: 2 points). SARC-F ≥ 4 is indicative of sarcopenia; SARC-CalF is based on SARC-F and incorporates calf circumference (men <34 cm, women <33 cm). A score of 10 is assigned if calf circumference is below the threshold; a score of 0 is assigned if it is above the threshold (Forsythe et al., 2020). Healthcare professionals can adjust treatment measures based on the assessment results and promptly implement intervention measures.

#### 5.2.7 Gender differences are also an important consideration that needs to be taken into account

It is worth noting that there may be differences in the occurrence of dermatomyositis between males and females due to the influence of sex hormones and the disparity in body composition, with different diagnostic cutoff points (Janssen et al., 2002; Sipilä et al., 2013; Grossmann, 2014; Sugioka et al., 2016). Gender influences the expression of PD-1 in tumors and the efficacy of ICIs. Firstly, studies have found that the incidence of lung cancer in females is continuously increasing, and female patients are more prone to develop adenocarcinoma compared to males. Inherent and adaptive differences in immune responses between males and females contribute to the gender-related disparity in the response to PD-1/PD-L1-dependent immunotherapy (Gu et al., 2022). Gu et al. have found that female lung cancer patients exhibit higher levels of sPD-1 in serum and PD-1 expression on CD4^+^ T cells compared to male patients (Gu et al., 2022). Another study has shown that male patients experience greater improvements in OS and PFS when receiving immune checkpoint inhibitor therapy compared to female patients (Wu et al., 2018). A meta-analysis has also demonstrated that anti-PD-1/anti-PD-L1 monotherapy is highly effective in male patients but not in female patients, even in NSCLC with high PD-L1 expression levels (Conforti et al., 2021). These research findings indicate the significant impact of gender on PD-1 expression in tumors and the efficacy of ICIs. Further investigation into the mechanisms underlying gender differences is of great significance for optimizing tumor treatment strategies and enhancing personalized therapeutic outcomes. Although there are currently no specific intervention measures and recommendations to consider the effect of gender on the efficacy of PD-1 inhibitors in dermatomyositis, understanding the influence of gender differences on dermatomyositis is important for personalized treatment and optimizing therapeutic efficacy. Future research may explore the impact of factors such as sex hormones on the pathogenesis of dermatomyositis and further investigate the role of gender in PD-1 inhibitor therapy. This will contribute to providing more effective treatment options and intervention measures for patients.

The above strategies need to be individualized based on the patient’s specific condition and treatment plan, in collaboration with the medical team and nutritionists, ensuring the safety and suitability of the strategies. Additionally, establishing positive communication and education with patients, encouraging their active participation in measures to prevent sarcopenia, and ensuring their understanding and compliance with the strategies are important. In conclusion, by early intervention and active management of sarcopenia, the impact on the efficacy of PD-1 inhibitors can be mitigated, improving treatment outcomes and survival rates for patients.

## 6 Conclusion

Sarcopenia may have a negative impact on the efficacy of PD-1 inhibitors in NSCLC patients. Current clinical studies have shown that NSCLC patients with sarcopenia exhibit poorer overall survival and progression-free survival during PD-1 inhibitor treatment. Sarcopenia may affect the therapeutic effects of PD-1 inhibitors through both immune and metabolic mechanisms. In the absence of sufficient preventive and treatment measures, pre-emptive interventions before PD-1 inhibitor treatment can be implemented, such as exercise and rehabilitation training, nutritional support, psychological support and rehabilitation psychology, as well as regular monitoring and assessment. In the future, further research can enhance our understanding of the mechanisms by which sarcopenia affects PD-1 inhibitors and explore strategies to overcome this impact. Firstly, investigating the mechanisms of inflammatory factors in sarcopenic patients, particularly those associated with the efficacy of PD-1 inhibitors, and intervening and regulating high levels of inflammatory markers in sarcopenic patients may improve the effectiveness of PD-1 inhibitors. Secondly, studying the impact of sarcopenia on energy metabolism, particularly in immune cells and tumor cells, and identifying targets in metabolic pathways may enhance the therapeutic effects of PD-1 inhibitors. Additionally, developing personalized treatment strategies based on the impact of sarcopenia on the efficacy of PD-1 inhibitors may be an important direction. Tailoring individualized treatment plans, including exercise and rehabilitation training, nutritional support, and psychological support, based on the degree of sarcopenia and relevant factors in patients, can enhance the efficacy of PD-1 inhibitors.
